# The Brief Case: A renal abscess caused by ST35-KL108, a strain of multidrug-resistant hypervirulent *Klebsiella pneumoniae*

**DOI:** 10.1128/jcm.02057-24

**Published:** 2025-05-14

**Authors:** Fang Qian, Dongyue Lyu, Jiazhen Guo, Ran Duan, Shuai Qin, Hanyu Sha, Huaiqi Jing, Xin Wang, Zhihai Chen

**Affiliations:** 1 National Key Laboratory of Intelligent Tracking and Forecasting for Infectious Diseases, Beijing Ditan Hospital, Capital Medical University 12517, Beijing, China; 2 National Institute for Communicable Disease Control and Prevention, Chinese Center for Disease Control and Prevention 96698, Beijing, China; Endeavor Health, Evanston, Chicago, Illinois, USA

**Keywords:** hypervirulent *Klebsiella pneumoniae*, renal abscess, ST35-KL108, multidrug-resistant, ESBL

## CASE

The patient was an 86-year-old woman with hypertension, type 2 diabetes mellitus, and coronary artery disease. She presented to the hospital with a high fever, reaching a maximum temperature of 39.3°C, along with an altered consciousness (Glasgow Coma Scale [GCS] score of 13/15 [E4V4M5]). The patient underwent a comprehensive blood test that included a complete blood count, liver function tests, and coagulation function tests. The results revealed a neutrophil count (NE#) of 7.07 × 10^9^ /L, a lymphocyte count (LY#) of 0.62 × 10^9^ /L, and a C-reactive protein (CRP) level of 164.8 mg/L, indicating significant inflammation. An abdominal computed tomography (CT) was also performed. The initial diagnosis included fever and impaired consciousness, sepsis, acute kidney damage, and cardiac dysfunction. Empirical antimicrobial therapy with meropenem (1 g intravenously q12h) was administered, along with diuretics for potassium reduction and nutritional support. On the second day after admission, CRP was as high as 106.4 mg/L, and procalcitonin levels were measured at 83.81 ng/mL. Blood cultures were performed, and on the fifth day, *Klebsiella pneumoniae* subsp. *pneumoniae* was isolated. Urine cultures were negative. The patient was continued on intravenous meropenem treatment.

**Fig 1:**
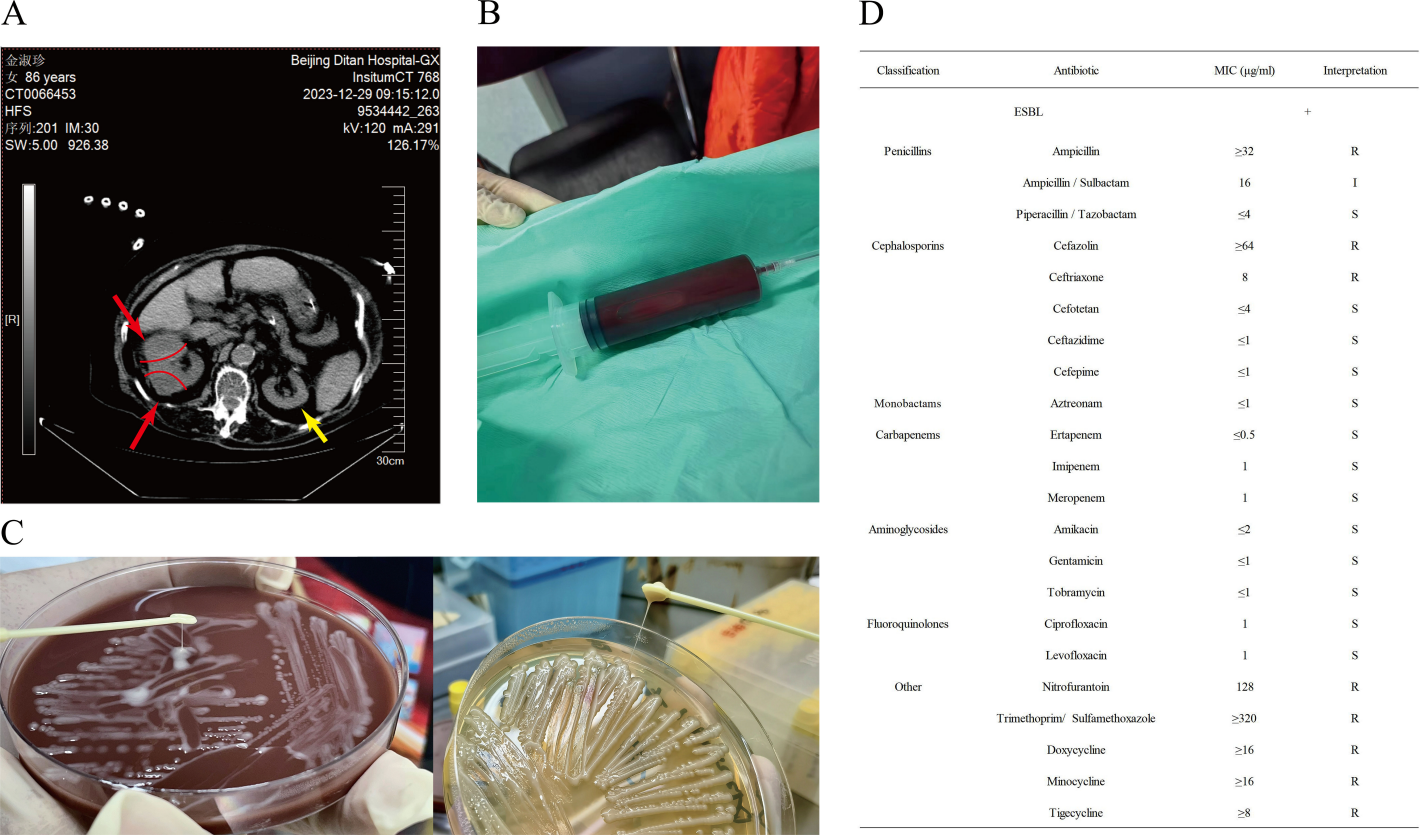
CT image, pus of the patient’s renal abscess and stringing test, antibiotic sensitivity of *K. pneumoniae.* (**A**) On the fifth day after admission, two clumpy abnormal soft tissue density shadows were seen in the right kidney that were larger than those observed on the day of admission, and the hyperdense exudative shadows are slightly absorbed. (**B**) Renal abscess pus was extracted by puncture on the 10th day after admission. (**C**) Strain DT20240104 JSZ. K.P.X on a chocolate agar plate and a Luria broth agar plate revealed the “stringing” phenomenon. (**D**) Antibiotic sensitivity of *K. pneumoniae.* Note: The red arrows and red lines indicate the location of pathological changes in the right kidney; the yellow arrow indicates the normal-sized left kidney. R, resistance; I, intermediate resistance; S, susceptibility; MIC, minimum inhibitory concentration; “+”, positive for extended-spectrum β-lactamases (ESBL) production.

Taking into account the patient’s clinical symptoms at disease onset were a high fever and altered consciousness, accompanied by a Sequential Organ Failure Assessment (SOFA) score of 4, sepsis was considered a possible diagnosis. This necessitated further investigation to ascertain the underlying pathogen and evaluate any potential organ impairment. To exclude the possibility of co-infection, metagenomic next-generation sequencing (mNGS) of plasma was conducted on the fourth day after admission, identifying *K. pneumoniae* with 320 reads (40.21%). Furthermore, the dynamic changes in the right renal CT provide a reliable imaging basis for the clinical diagnosis of renal abscess, as detailed below. An abdominal CT conducted on the fifth day after admission revealed two mass-shaped lesions in the right kidney, which have expanded compared with the lesions on the day of admission, with partial absorption of the high-density exudative shadows (Fig. 1A). On the 10th day after admission, the patient underwent ultrasound-guided percutaneous drainage of her renal abscess using a pigtail catheter. The procedure revealed the presence of sanguinopurulent fluid (Fig. 1B), which tested positive on the Rivalta test. This result indicates that the fluid is classified as an exudate, thereby suggesting the presence of an inflammatory process. *K. pneumoniae* (DT20240104 JSZ.K.p.X, Genbank accession no. CP158191.1, no. CP158192.1, and no. CP158193.1) was isolated from aspirated fluid and had a positive string test result (Fig. 1C). The isolate was an (extended-spectrum β-lactamases) ESBL-producing and multidrug-resistant (MDR) (1) hypervirulent *K. pneumoniae* (hvKp). Drug susceptibility testing, with the standards released by the Clinical and Laboratory Standards Institute (CLSI), revealed that the isolate was susceptible to meropenem and levofloxacin (Fig. 1D); therefore, the treatment regimen with meropenem was maintained. After the initiation of meropenem therapy, the patient’s peak temperature began to decline, and her mental status improved, indicating a positive response to treatment. On the 12th day after admission, intravenous meropenem dosing was increased to 1 g q8h due to the recovery of renal function. Follow-up imaging studies conducted on the 15th and 30th days after admission revealed a significant reduction in the size of the right renal kidney. The patient was treated with meropenem for 40 days, which was the entire duration of hospitalization. The patient’s body temperature and consciousness normalized, allowing for her discharge from the hospital. Upon discharge, she was prescribed levofloxacin orally for an additional week of therapy (to complete a total of 47 days of therapy). On day 12 post-discharge, the patient was seen in follow-up and continued to have no symptoms. Moreover, a repeat abdominal CT revealed at that time that the abscess lesions in the right kidney were essentially completely resolved.

## DISCUSSION

*K. pneumoniae* is an opportunistic pathogen associated with hospital- and community-acquired infections (2–4). Classic *K. pneumoniae* (cKp) and hvKp exhibit distinct clinical and laboratory characteristics. CKp infections are predominantly nosocomial, primarily affecting immunocompromised patients in hospital settings, particularly the elderly and those in intensive care units. These infections typically involve a single organ system, such as the urinary tract, bloodstream, abdomen, or wound sites. Affected individuals often have pre-existing medical conditions or a history of invasive procedures, including prolonged catheterization or mechanical ventilation (5). Conversely, hvKp infections are frequently community-acquired and can lead to invasive disease in both immunocompetent and immunocompromised patients, as well as serious infections in healthy people. HvKp is characterized by its propensity for metastatic spread, rapid disease progression, and a significantly elevated risk of morbidity and mortality if not treated promptly (6, 7). HvKp is the primary pathogen responsible for *K. pneumoniae*-induced liver abscess and can cause serious infections in healthy individuals. This pathogen has the potential to disseminate systemically, affecting multiple organ systems and resulting in pneumonia, endophthalmitis, meningitis, or necrotizing fasciitis (8–10). In clinical microbiology laboratories, hvKp represents a more pathogenic variant of *K. pneumoniae*. This variant is commonly identified through the application of the string test, detection of virulence genes, and capsular serotyping. In recent decades, hvKp has attracted significant attention, especially in the Asia-Pacific region (11), where the incidence of hvKp infections has notably increased, showing a trend toward hospital-acquired infections and an increase in antibiotic resistance (12).

This is the report of a patient with a hvKp-associated unilateral and deep abscess renal abscess with multiple lesions. The blood culture results indicated that the sample from the second day tested positive for *K. pneumoniae*, whereas the subsequent samples, after treatment, showed no bacterial growth. This suggests that the bacteria in the bloodstream were gradually eliminated, resulting in a low quantity of nucleic acids in the sample for mNGS. The mNGS results showed a positive detection for the *Klebsiella* genus as positive (530 reads, 66.58%), of which 320 reads (40.21%) were identified as *K. pneumoniae*. The remaining reads primarily originated from host DNA. The consistency between the two test results indicated a bloodstream infection with *K. pneumoniae*. The patient had no history of chronic urinary tract infection, and both renal ultrasound and CT showed no evidence of reflux nephropathy from urinary calculi, hydronephrosis, or a chronic renal scar after admission. No bacteria were found in the patient’s urine smear and culture. Thus, it was assumed that the renal abscess developed from hematogenous dissemination. Renal abscesses caused by hematogenous bacterial dissemination are relatively rare and are more common in *Staphylococcus*-associated infections of the renal cortex (13, 14). The symptoms of renal abscess are often non-specific (15), and the CT scan contributed to this timely diagnosis. The scan on day 5 demonstrated an enlargement of the lesion, suggesting a worsening infection and significant invasiveness. The imaging feature that hyperdense exudative shadows were partially absorbed in the lesion indicated that the inflammatory exudate underwent necrotic liquefaction and transformed into pus, consistent with the clinicopathological process of renal abscesses.

Most studies define a putative hvKp infection based on clinical features and/or a positive string test (16). Although a majority of hvKp isolates present as hypermucoviscous (positive string test), this characteristic is not present in all hvKp, and some cKp can present this phenotype (17, 18). Consequently, it is imperative to perform comprehensive assessments of virulence genes to identify hvKp accurately. The identification of genes such as *peg-344*, *iroB*, *iucA*, and *rmpA* is instrumental in detecting hvKp (4, 19). Clinical laboratories may use techniques like PCR to identify these genes rapidly. In this case, whole genome sequencing (WGS) was performed on the strain (DT20240104 JSZ.K.p.X) to gather additional information. It was a ST35-KL108 hvKp with a lipopolysaccharide serotype (O antigen) of O1 (20). Except for *iucA*, the genes *peg-344*, *iroB*, and *rmpA* are all present in the strain DT20240104 JSZ.K.p.X. The *rmpA* gene, which is rarely found in cKp but highly prevalent in hvKp, gives the bacterium a highly mucoid phenotype by greatly increasing the formation of the capsule (21) and, in concert with fimbriae and siderophores (such as enterobactin and salmochelin) (22), propels hvKp to cause invasive disease. *Peg-344* holds potential application value in the rapid diagnosis of hvKp (23). Sixty-nine resistance genes located on the chromosome and plasmid 2 were predicted, and siderophore, fimbriae, and other related virulence genes were detected on the chromosome. The commonly detected types of hvKp are ST23-K1 and ST86-K2 (24), which are usually associated with serious human infections (25) and reported from China, Russia, and Japan (26–28). As evidenced by this case, the rare ST35 multilocus sequence type (MLST) and KL108 capsular type (29) strain DT20240104 JSZ.K.p.X demonstrated significant virulence. Therefore, although identifying the subtype of *K. pneumoniae* plays a crucial role in the rapid preliminary assessment of their virulence potential, a comprehensive evaluation of various factors is essential for accurately determining the actual virulence of these strains. Rare high-virulence strains should not be overlooked, and subtype tests should be improved to provide a complete and thorough understanding of the virulence.

In recent decades, there has been an increase in the acquisition of resistance to a wide range of antibiotics by strains derived from the cKp. As a result, the World Health Organization listed *K. pneumoniae* as a priority pathogen in 2024 (30, 31). Compared with cKp, hvKp is more likely to retain antimicrobial susceptibility due to the greater difficulty in integrating antimicrobial resistance plasmids (32). However, in the face of antibiotic selection pressure, there are increasing reports of hvKp possessing ESBL, carbapenemase, and even colistin-resistant plasmids (33, 34). In a study by Liu C et al. (12) involving 79 hvKp strains, 31 (43.1%) hospital and healthcare-associated hvKp isolates were MDR, 30 (41.7%) expressed ESBLs, and 16 (22.2%) exhibited carbapenem resistance; none of the seven community-acquired hvKp isolates showed MDR or expressed ESBLs. The study in South and Southeast Asia showed that 7% of the strains simultaneously carry the *iuc* gene and ESBL and/or carbapenemase genes, demonstrating the coexistence of resistance genes and virulence genes (35). These resistance patterns create greater challenges for detection in clinical microbiology laboratories, complicating the clinical management of hvKp infections. Choosing an effective empirical regimen will become more difficult, requiring accurate identification of hvKp resistance through routine antimicrobial susceptibility testing and molecular biological detection methods, such as PCR and whole-genome sequencing. This is crucial for selecting appropriate antibiotic treatment regimens, improving patient cure rates, and controlling the spread of resistant strains.

The identification of hvKp is critically important for patient management. Improving the quality of clinical microbiological testing and accurately diagnosing hvKp facilitate the selection of appropriate anti-infective treatment strategies and the implementation of surgical drainage when indicated, both of which can significantly influence the prognosis of hvKp infections (36). Furthermore, through the application of isolation protocols and environmental disinfection, the transmission of infection can be effectively mitigated, thereby decreasing the likelihood of cross-infection (37). Additionally, clarifying the nature of the infection enables healthcare professionals to closely monitor the patient’s condition and provide tailored recovery recommendations, ultimately enhancing the patient’s prognosis and minimizing the risk of complications. In cases where patients exhibit atypical symptoms indicative of hvKp infection, it is essential to maintain a heightened level of vigilance and initiate prompt diagnostic procedures.

## SELF-ASSESSMENT QUESTIONS

1. Currently, which is the most common infection caused by hypervirulent *K. pneumoniae*?
  - Liver abscesses
  - Renal abscess
  - Pneumonia
  - Endophthalmitis
2. Which of the following methods helps the least in identifying hypervirulent *K. pneumoniae*?
  - String test
  - Serotype and capsule type
  - Sequencing-based technologies
  - Antimicrobial susceptibility test
3. Which type of antibiotic has seen a significant increase in resistance among *K. pneumoniae* in recent years, posing a major challenge to global public health?
  - Macrolides (such as erythromycin)
  - Aminoglycosides (such as gentamicin)
  - Carbapenems (such as imipenem)
  - Sulfonamides (such as sulfamethoxazole)

## ANSWERS TO SELF-ASSESSMENT QUESTIONS

1. Currently, which is the most common abscess caused by hypervirulent *K. pneumoniae*?
  - Liver abscesses
  - Renal abscess
  - Pneumonia
  - Endophthalmitis

Answer: A.

Among the infections caused by hypervirulent *K. pneumoniae*, liver abscesses are currently the most commonly observed. Other possible infections include renal abscesses, pneumonia, and endophthalmitis.

- Which of the following methods helps the least in identifying hypervirulent *K. pneumoniae*?
  - String test
  - Serotype and capsule type
  - Sequencing-based technologies
  - Antimicrobial susceptibility test

Answer: D.

To accurately identify hypervirulent strains of *K. pneumoniae*, various methods can be utilized, including the string test, serotyping and capsular typing, and detection of specific virulence genes. Combining these methods provides a comprehensive approach to identifying hypervirulent *K. pneumoniae*. Antimicrobial susceptibility testing can be beneficial in guiding clinical treatment; however, it is not the primary method for the identification of hvKp.

- Which type of antibiotic has seen a significant increase in resistance among *K. pneumoniae* in recent years, posing a major challenge to global public health?
  - Macrolides (such as erythromycin)
  - Aminoglycosides (such as gentamicin)
  - Carbapenems (such as imipenem)
  - Sulfonamides (such as sulfamethoxazole)

Answer: C.

The resistance of *K. pneumoniae* to carbapenem antibiotics has risen significantly in recent years. As a class of broad-spectrum antibiotics, carbapenems are often regarded as the last line of defense for treating severe infections. However, the continuous increase in resistance poses a serious threat to the efficacy of these vital medications. According to CHINET data, the resistance rate of *K. pneumoniae* to imipenem has surged by approximately 20% between 2005 and 2022 (38). Although macrolides, aminoglycosides, and sulfonamides are also commonly used antibiotics, the resistance of *K. pneumoniae* to these agents has not escalated significantly in recent years compared with the alarming trends observed with carbapenems.

**TAKE-HOME POINTS**

- Hypervirulent K. pneumoniae is a strain characterized by a high mucoid phenotype and various virulence factors. Compared with classical Klebsiella pneumoniae, it has a stronger virulence and can cause severe disseminated infections in immunocompetent hosts.
- Clinical microbiology laboratories can perform preliminary screening through the mucoid string test and further identify hvKp strains using techniques such as PCR to detect virulence genes (e.g., rmpA and iucA) or whole genome sequencing (WGS).
- Accurate identification of hvKp is crucial for the early diagnosis and precise treatment of patients, aiding in the selection of appropriate antibiotic regimens, reducing mortality rates, and preventing the spread of nosocomial infections.
- Due to the rapid dissemination and increasing drug resistance of hvKp, timely identification and isolation of infected patients are key to controlling the infection. Additionally, personalized treatment strategies should be developed based on the characteristics of the strain to improve patient outcomes.

## Data Availability

The genome sequence in this study is available in GenBank (accession nos. CP158191.1, CP158192.1, and CP158193.1).

## References

[1] Magiorakos A-P, Srinivasan A, Carey RB, Carmeli Y, Falagas ME, Giske CG, Harbarth S, Hindler JF, Kahlmeter G, Olsson-Liljequist B, Paterson DL, Rice LB, Stelling J, Struelens MJ, Vatopoulos A, Weber JT, Monnet DL. 2012. Multidrug-resistant, extensively drug-resistant and pandrug-resistant bacteria: an international expert proposal for interim standard definitions for acquired resistance. Clin Microbiol Infect 18:268–281. doi:10.1111/j.1469-0691.2011.03570.x 21793988

[2] Wyres KL, Holt KE. 2018. Klebsiella pneumoniae as a key trafficker of drug resistance genes from environmental to clinically important bacteria. Curr Opin Microbiol 45:131–139. doi:10.1016/j.mib.2018.04.004 29723841

[3] Clegg S, Murphy CN. 2016. Epidemiology and virulence of Klebsiella pneumoniae. Microbiol Spectr 4. doi:10.1128/microbiolspec.UTI-0005-2012 26999397

[4] Russo TA, Olson R, Fang C-T, Stoesser N, Miller M, MacDonald U, Hutson A, Barker JH, La Hoz RM, Johnson JR. 2018. Identification of biomarkers for differentiation of hypervirulent Klebsiella pneumoniae from classical K. pneumoniae. J Clin Microbiol 56:e00776-18. doi:10.1128/JCM.00776-18 29925642 PMC6113484

[5] Zhu W-M, Yuan Z, Zhou H-Y. 2020. Risk factors for carbapenem-resistant Klebsiella pneumoniae infection relative to two types of control patients: a systematic review and meta-analysis. Antimicrob Resist Infect Control 9:23. doi:10.1186/s13756-020-0686-0 32005246 PMC6995231

[6] Lee J, Hwang J-H, Yeom JH, Lee S, Hwang J-H. 2024. Analysis of virulence profiles in clinical isolates of Klebsiella pneumoniae from renal abscesses: clinical significance of hypervirulent isolates. Front Cell Infect Microbiol 14:1367111. doi:10.3389/fcimb.2024.1367111 38606296 PMC11007163

[7] Lin Y-T, Jeng Y-Y, Chen T-L, Fung C-P. 2010. Bacteremic community-acquired pneumonia due to Klebsiella pneumoniae: clinical and microbiological characteristics in Taiwan, 2001-2008. BMC Infect Dis 10:307. doi:10.1186/1471-2334-10-307 20973971 PMC2987304

[8] Hassanin F, Khawjah D, Elkhamary S, Al Hussain H. 2021. Renal abscesses and endogenous endophthalmitis due to hypermucoviscous hypervirulent Klebsiella pneumoniae (HVKP). IDCases 24:e01130. doi:10.1016/j.idcr.2021.e01130 33996464 PMC8094904

[9] Fang C-T, Lai S-Y, Yi W-C, Hsueh P-R, Liu K-L, Chang S-C. 2007. Klebsiella pneumoniae genotype K1: an emerging pathogen that causes septic ocular or central nervous system complications from pyogenic liver abscess. Clin Infect Dis 45:284–293. doi:10.1086/519262 17599305

[10] Liu YC, Cheng DL, Lin CL. 1986. Klebsiella pneumoniae liver abscess associated with septic endophthalmitis. Arch Intern Med 146:1913–1916. 3532983

[11] Shon AS, Bajwa RPS, Russo TA. 2013. Hypervirulent (hypermucoviscous) Klebsiella pneumoniae: a new and dangerous breed. Virulence 4:107–118. doi:10.4161/viru.22718 23302790 PMC3654609

[12] Liu C, Du P, Xiao N, Ji F, Russo TA, Guo J. 2020. Hypervirulent Klebsiella pneumoniae is emerging as an increasingly prevalent K. pneumoniae pathotype responsible for nosocomial and healthcare-associated infections in Beijing, China. Virulence 11:1215–1224. doi:10.1080/21505594.2020.1809322 32921250 PMC7549996

[13] Lee BE, Seol HY, Kim TK, Seong EY, Song SH, Lee DW, Lee SB, Kwak IS. 2008. Recent clinical overview of renal and perirenal abscesses in 56 consecutive cases. Korean J Intern Med 23:140–148. doi:10.3904/kjim.2008.23.3.140 18787367 PMC2686968

[14] Zhan Z, Lin X, Li G, Zeng J, Su D, Liao J, Shen Q. 2023. Renal abscess complicating acute pyelonephritis in children: two cases report and literature review. Medicine (Baltimore) 102:e36355. doi:10.1097/MD.0000000000036355 38050281 PMC10695508

[15] Liu X-Q, Wang C-C, Liu Y-B, Liu K. 2016. Renal and perinephric abscesses in West China Hospital: 10-year retrospective-descriptive study. World J Nephrol 5:108–114. doi:10.5527/wjn.v5.i1.108 26788470 PMC4707163

[16] Fang C-T, Chuang Y-P, Shun C-T, Chang S-C, Wang J-T. 2004. A novel virulence gene in Klebsiella pneumoniae strains causing primary liver abscess and septic metastatic complications. J Exp Med 199:697–705. doi:10.1084/jem.20030857 14993253 PMC2213305

[17] Catalán-Nájera JC, Garza-Ramos U, Barrios-Camacho H. 2017. Hypervirulence and hypermucoviscosity: Two different but complementary Klebsiella spp. phenotypes? Virulence 8:1111–1123. doi:10.1080/21505594.2017.1317412 28402698 PMC5711391

[18] Parrott AM, Shi J, Aaron J, Green DA, Whittier S, Wu F. 2021. Detection of multiple hypervirulent Klebsiella pneumoniae strains in a New York City hospital through screening of virulence genes. Clin Microbiol Infect 27:583–589. doi:10.1016/j.cmi.2020.05.012 32461145

[19] Russo TA, Marr CM. 2019. Hypervirulent Klebsiella pneumoniae. Clin Microbiol Rev 32:e00001-19. doi:10.1128/CMR.00001-19 31092506 PMC6589860

[20] Bulati M, Busà R, Carcione C, Iannolo G, Di Mento G, Cuscino N, Di Gesù R, Piccionello AP, Buscemi S, Carreca AP, Barbera F, Monaco F, Cardinale F, Conaldi PG, Douradinha B. 2021. Klebsiella pneumoniae lipopolysaccharides serotype O2afg induce poor inflammatory immune responses ex vivo. Microorganisms 9:1317. doi:10.3390/microorganisms9061317 34204279 PMC8234205

[21] Surgers L, Boyd A, Girard P-M, Arlet G, Decré D. 2016. ESBL-producing strain of hypervirulent Klebsiella pneumoniae K2, France. Emerg Infect Dis 22:1687–1688. doi:10.3201/eid2209.160681 27532217 PMC4994372

[22] Alcántar-Curiel MD, Blackburn D, Saldaña Z, Gayosso-Vázquez C, Iovine NM, De la Cruz MA, Girón JA. 2013. Multi-functional analysis of Klebsiella pneumoniae fimbrial types in adherence and biofilm formation. Virulence 4:129–138. doi:10.4161/viru.22974 23302788 PMC3654611

[23] Liu C, Guo J. 2019. Hypervirulent Klebsiella pneumoniae (hypermucoviscous and aerobactin positive) infection over 6 years in the elderly in China: antimicrobial resistance patterns, molecular epidemiology and risk factor. Ann Clin Microbiol Antimicrob 18:4. doi:10.1186/s12941-018-0302-9 30665418 PMC6341648

[24] Siu LK, Yeh K-M, Lin J-C, Fung C-P, Chang F-Y. 2012. Klebsiella pneumoniae liver abscess: a new invasive syndrome. Lancet Infect Dis 12:881–887. doi:10.1016/S1473-3099(12)70205-0 23099082

[25] Wyres KL, Wick RR, Gorrie C, Jenney A, Follador R, Thomson NR, Holt KE. 2016. Identification of Klebsiella capsule synthesis loci from whole genome data. Microb Genom 2:e000102. doi:10.1099/mgen.0.000102 28348840 PMC5359410

[26] Fursova AD, Fursov MV, Astashkin EI, Novikova TS, Fedyukina GN, Kislichkina AA, Alexandrova IA, Ershova ON, Dyatlov IA, Fursova NK. 2021. Early response of antimicrobial resistance and virulence genes expression in classical, hypervirulent, and hybrid hvKp-MDR Klebsiella pneumoniae on antimicrobial stress. Antibiotics (Basel) 11:7. doi:10.3390/antibiotics11010007 35052884 PMC8773033

[27] Kikuchi S, Kosai K, Ota K, Mitsumoto-Kaseida F, Sakamoto K, Hasegawa H, Izumikawa K, Mukae H, Yanagihara K. 2023. Clinical and microbiological characteristics of bloodstream infection caused by Klebsiella pneumoniae harboring rmpA in Japanese adults. Sci Rep 13:6571. doi:10.1038/s41598-023-33265-1 37085513 PMC10121676

[28] Pei N, Liu X, Jian Z, Yan Q, Liu Q, Kristiansen K, Li J, Liu W. 2023. Genome sequence and genomic analysis of liver abscess caused by hypervirulent Klebsiella pneumoniae. 3 Biotech 13:76. doi:10.1007/s13205-023-03458-6 PMC9898476 36748017

[29] Kasimova AA, Shneider MM, Evseev PV, Shelenkov AA, Mikhailova YV, Miroshnikov KA, Chebotar IV, Shagin DA. 2023. The structure of Klebsiella pneumoniae K108 capsular polysaccharide is similar to Escherichia coli colanic acid. Int J Biol Macromol 244:125403. doi:10.1016/j.ijbiomac.2023.125403 37330077

[30] World Health Organisation. 2024. Pathogens Prioritization: A Scientific Framework for Epidemic and Pandemic Research Preparedness. https://www.who.int/publications/m/item/pathogens-prioritization-a-scientific-framework-for-epidemic-and-pandemic-research-preparedness.

[31] World Health Organisation. 2024. Antimicrobial Resistance, Hypervirulent Klebsiella Pneumoniae - Global Situation. https://www.who.int/emergencies/disease-outbreak-news/item/2024-DON527.

[32] Lam MMC, Wyres KL, Duchêne S, Wick RR, Judd LM, Gan Y-H, Hoh C-H, Archuleta S, Molton JS, Kalimuddin S, Koh TH, Passet V, Brisse S, Holt KE. 2018. Population genomics of hypervirulent Klebsiella pneumoniae clonal-group 23 reveals early emergence and rapid global dissemination. Nat Commun 9:2703. doi:10.1038/s41467-018-05114-7 30006589 PMC6045662

[33] Taraghian A, Nasr Esfahani B, Moghim S, Fazeli H. 2020. Characterization of hypervirulent extended-spectrum β-Lactamase-producing Klebsiella pneumoniae among urinary tract infections: the first report from Iran. Infect Drug Resist 13:3103–3111. doi:10.2147/IDR.S264440 32982325 PMC7489934

[34] Aslam B, Khurshid M, Ahmad M, Baloch Z. 2022. A response to article “Distribution of mcr-1 harboring hypervirulent Klebsiella pneumoniae in clinical specimens and lytic activity of bacteriophage KpnM against isolates”. Infect Drug Resist 15:6601–6602. doi:10.2147/IDR.S394683 36386412 PMC9665074

[35] Wyres KL, Nguyen TNT, Lam MMC, Judd LM, van Vinh Chau N, Dance DAB, Ip M, Karkey A, Ling CL, Miliya T, Newton PN, Lan NPH, Sengduangphachanh A, Turner P, Veeraraghavan B, Vinh PV, Vongsouvath M, Thomson NR, Baker S, Holt KE. 2020. Genomic surveillance for hypervirulence and multi-drug resistance in invasive Klebsiella pneumoniae from South and Southeast Asia. Genome Med 12:11. doi:10.1186/s13073-019-0706-y 31948471 PMC6966826

[36] Li L, Yuan Z, Chen D, Xie X, Zhang B. 2020. Clinical and microbiological characteristics of invasive and hypervirulent Klebsiella pneumoniae infections in a teaching hospital in China. Infect Drug Resist 13:4395–4403. doi:10.2147/IDR.S282982 33328744 PMC7734077

[37] Marr CM, Russo TA. 2019. Hypervirulent Klebsiella pneumoniae: a new public health threat. Expert Rev Anti Infect Ther 17:71–73. doi:10.1080/14787210.2019.1555470 30501374 PMC6349525

[38] Qin X, Ding L, Hao M, Li P, Hu F, Wang M. 2024. Antimicrobial resistance of clinical bacterial isolates in China: current status and trends. JAC-Antimicrobial Resistance 6. doi:10.1093/jacamr/dlae052 PMC10977948 38549710

